# The role of social network analysis as a learning analytics tool in online problem based learning

**DOI:** 10.1186/s12909-019-1599-6

**Published:** 2019-05-22

**Authors:** Mohammed Saqr, Ahmad Alamro

**Affiliations:** 1 School of Computing, University of Eastern Finland, Joensuu Campus, Yliopistokatu 2, P.O. Box 111, fi-80100 Joensuu, Finland; 2 Department of Computer and System Sciences (DSV), Stockholm University, Borgarfjordsgatan 12, PO Box 7003, SE-164 07 Kista, Sweden; 3 College of Medicine, Qassim University, Qassim, Kingdom of Saudi Arabia

**Keywords:** Social network analysis, problem-based learning, Blended learning, blended problem-based learning, Learning analytics

## Abstract

**Background:**

Social network analysis (SNA) might have an unexplored value in the study of interactions in technology-enhanced learning at large and in online (Problem Based Learning) PBL in particular. Using SNA to study students’ positions in information exchange networks, communicational activities, and interactions, we can broaden our understanding of the process of PBL, evaluate the significance of each participant role and learn how interactions can affect academic performance.

The aim of this study was to study how SNA visual and mathematical analysis can be sued to investigate online PBL, furthermore, to see if students’ position and interaction parameters are associated with better performance.

**Methods:**

This study involved 135 students and 15 teachers in 15 PBL groups in the course of “growth and development” at Qassim University. The course uses blended PBL as the teaching method. All interaction data were extracted from the learning management system, analyzed with SNA visual and mathematical techniques on the individual student and group level, centrality measures were calculated, and participants’ roles were mapped. Correlation among variables was performed using the non-parametric Spearman rank correlation test.

**Results:**

The course had 2620 online interactions, mostly from students to students (89%), students to teacher interactions were 4.9%, and teacher to student interactions were 6.15%. Results have shown that SNA visual analysis can precisely map each PBL group and the level of activity within the group as well as outline the interactions among group participants, identify the isolated and the active students (leaders and facilitators) and evaluate the role of the tutor. Statistical analysis has shown that students’ level of activity (outdegree r_s_(133) = 0.27, *p* = 0.01), interaction with tutors (r_s_ (133) = 0.22, *p* = 0.02) are positively correlated with academic performance.

**Conclusions:**

Social network analysis is a practical method that can reliably monitor the interactions in an online PBL environment. Using SNA could reveal important information about the course, the group, and individual students. The insights generated by SNA may be useful in the context of learning analytics to help monitor students’ activity.

## Background

Social constructivists view learning as an active construction of knowledge that occurs through social interaction and dialogue among learners. Interaction becomes particularly meaningful when learners are coached in a positive atmosphere and when the students have the chance to argue, debate, and offer alternate perspectives or contribute to ideas [1, 2]. Aiming to harness the potentials of the approach, the social constructivist methods have been embraced by several modern pedagogies such as problem and team-based learning that encourage collaboration and promote meaningful interactions among learners. Problem-based learning (PBL) uses problems as triggers to facilitate discourse and interaction among students, the discussions occur in small groups and are facilitated by teachers [3]. The PBL process is structured in a way to help students elaborate and activate previous information [3, 4].

Online problem-based learning requires students to engage in active discussions in two types of dialogical spaces; the content and the relational spaces. Learners interact in the content space towards the goal of acquiring a deeper understanding of the domain of knowledge; activities include collecting information, discussing concepts and proposing solutions to the problem [5–7]. Communicative activities in the relational space deal with the interpersonal relations and interactions among collaborators. The primary goal of these interactions is to reach a common understanding of the concepts and content under discussion [5, 8].

There is overwhelming evidence of the value of learners’ interaction with peers, teachers, and the content, which is widely recognized as a pivotal ingredient of modern education [9, 10]. However, offering the learners the opportunity to interact does not directly translate to effective interactions and working online together does not mean collaboration [6, 11, 12]. Moreover, online collaboration requires instructional support, scaffolding by teachers, active coordinating of collaboration dynamics, engagement of learners in a stimulating environment [11, 13], which begs the need for a mechanism to monitor the efficiency of online interactions and design a data-driven intervention that supports effective collaboration.

## Social network analysis

Social network analysis (SNA) is a collection of methods and tools that could be used to study the relationships, interactions and communications. As such, SNA is suitable for the study and possibly monitoring of online interactions as it can automatically analyze interaction data, bringing a bird-eye view of the group social structure, the interaction patterns, as well as the mapping of all communications in the relational space [14–17]. SNA can be implemented in two main ways, visualization, and mathematical analysis.

SNA visualization renders relationships between actors in social networks by graphs known as sociograms; the sociogram portrays actors (nodes in SNA terms) as points, and relationships (edges in SNA terms) as arrows originating from the source of the interaction and pointing to the target of interaction [17]. SNA may help visualize the interactions among participants and may reveal who are the important actors in the interactions and who are the isolated actors, what are the groups that shows dense interactions or sparse interactions that may need support. It may also reveal the active moderators who are participating and interacting with students, and the extent of their interactions. PBL is a collaborative learning where small groups interdependently work together and as such evaluation of collaborative interactions is important.

The SNA mathematical analysis quantifies network parameters on individual actor levels, as well as group level. The mathematical analysis of SNA uses graph theory concepts to calculate metrics representing the nodes, the links or the network. Such as the distance to other actors in the network, the number of interactions with other actors, or how many times it bridged interactions between communities. These metrics are important in quantifying interactions, ranking nodes, or relationships. Parameters calculated at the actor level are called centrality scores, these scores are measures of the node position or importance in the social network. Since there are different contexts and probably different ways to consider the role important, there are different centrality scores. The measures chosen for this study are centrality measures of role and position in information exchange [17]. For example: number of received interactions are quantified as in-degree centrality, number of contributed interactions is called out-degree centrality. These parameters can add to our portfolio of information about students and might extend to learning analytics [14, 15]. In the context of PBL, one would be interested in the interactivity of participants, and their groups, and how these metrics could tell us about online PBL.

Previous research has indicated the utility of SNA in the study of collaborative learning; J Zhang and J Zhang [18] used SNA to diagnose gaps in problem solving and knowledge construction. Their findings highlighted problems such as students with low levels of interaction, the presence of few central students to the exchange of information. A Bakharia and S Dawson [14] have shown how to use SNA to map the online interactions among groups of students and identify the communities of collaborators. They were also able to recognize influential and isolated students who might need to be stimulated or supported. M Saqr, U Fors and M Tedre [19] used social network analysis to monitor collaborative groups and create a data-driven intervention that results in a meaningful improvement in the way students collaborate. Previous research results in learning analytics are promising, and some studies have given an indication of the potential of SNA centrality measures in predicting students’ performance [20, 21].

There is an apparent gap of the studies about the interactions in online PBL, particularly the relational and communicative activities. Most studies about the interactions in PBL targeted the content dimension through effortful resource intensive methods such as interviewing the learners, recording, and coding of PBL sessions or text mining techniques. Other researchers have used indirect examination and exploration using surveys and open-ended questionnaires [7, 22]. Such methods are practically demanding as well as challenging to standardize and difficult to implement by instructors [23, 24]. It is reasonable to argue that there is a need for mechanisms to ensure that online PBL meets the required curricular goals and objective and is actually collaborative. The methods need to be automatic, easy to implement and offer meaningful information that can help stakeholders improve the PBL process. In this study, we investigate the use the potentials of SNA in supporting learning and teaching. We have considered both visualization and mathematical analysis. Since the PBL process has three important aspects: the student, the actor and the group, we have used these aspects as the units of analysis.

The research question of this study can be formulated as follows:

- What can SNA visual and mathematical analysis tell about PBL?
- What interaction parameters as measured by SNA associated with better performance?

## Methods

### Design

The study was designed as an exploratory case study; a case study design allows an in-depth investigation of a case (Online PBL course in our study) from different perspectives.

### Participants

The study included data from 135 first year students (42 females and 93 males) and 15 tutors (10 males and 5 females), three students did not sign the ethical approval and 11 students dropped out of the course and so their data were not included. The course chosen was the Growth and Development of the year 2017, attended by first year students, the course is the third course in the year. The course covers issues of growth and development from different aspects, including anatomy, physiology and embryology. By the third course, students should have acquired a fair experience of the educational system and practiced online PBL for three months. The course duration is around 45 days, within them four weeks of PBL sessions were completed by all students.

### Setting

Qassim College of Medicine, Saudi Arabia has introduced PBL since 2001. The college adopted the seven jumps approach, student is expected to attend two sessions physically, one at the beginning of the week and another at the end, in between these sessions, students are offered an online forum where they continue to interact. The interactions start on the first day of the week by posting the learning issues of the weekly problem. Students are encouraged to discuss the learning issues, share information, collaboratively construct the required knowledge and work towards achieving the goals and the objectives of the PBL problem. By the last day of the week, each group is expected to wrap up what they have learnt together. The online discussions are assigned to the same students in the physical group and facilitated by the same tutors [7].

### Measures

The PBL represents the backbone of the educational system at the medical college and the objectives of the PBL sessions are written to cover the course objectives. The lectures, seminars and other educational activities are designed to support students understand the problem used in the PBL. The students are evaluated by multiple choice questions (MCQ), modified essay questions (MEQs) and Short-answer Essay Questions (SEQ) as well as practical exams. The grades considered for this study are only the sum of MCQ, MEQ and SEQ as they are designed to test students’ knowledge acquisition of PBL objectives and the exam blueprint is designed according to these objectives. The mean difficulty index of MCQs was 0.67, the mean discrimination index was 0.32 and the reliability index was 89.2%.

### Data collection

Interaction data were extracted from the learning management system. Custom Structured Query Language (SQL) queries were used to extract interaction data along with attributes of students and properties of posts, the data extracted for each post were the id of the sender, the id of the receiver, time the post was written, time it was modified, the content of the post, and forum and thread IDs. For each user we extracted user Id, role, PBL group and the posts he contributed. All participant IDs were removed to protect their confidentiality and re-coded, so the students labeled as S1 to S135 and the tutors were recoded as T1 to T15.

### SNA analysis

The analysis of SNA was done using Gephi version 9.2. Gephi is an open source application that has the capability to visually and mathematically analyze social networks through a graphical user interface. It can import a range of formats as well as export analysis data in an easy to use format for analysis [25].

### SNA visual analysis

The course network, as well as group networks, were analyzed and studied visually. Visualization was implemented to describe the general properties of the course network, such as the interactivity level, participants’ contributions, who is participating and identify the types of communications. The same was performed for each PBL group with special emphasis on the student-student and student tutor communications.

### SNA mathematical analysis

Mathematical analysis of participants’ interactivity may shed lights on important aspects such as interactivity and information exchange. As such, mathematical analysis was done at two levels, Individual students’ level (student and tutor), and group level, since they are the important factors in the PBL process.

#### Student level

For each student, we collected the following parameters: Type of interactions (student-student, student-tutor, and tutor-tutor). We also calculated centrality measures that are relevant to interactions and role in information exchange:

- Indegree centrality: the number of posts that are directed to a user, such as replies. A user receives more replies to contribution if the contribution is noteworthy, useful or arguable. As such in-degree centrality in this context reflect the importance of a user contribution as voted by other users. Out-degree centrality: the frequency of posts made by the user and reflects the activity of the user in the discussions.
- Degree centrality: is the sum of indegree and outdegree centralities and reflects the overall activity of the user
- Betweenness centrality: is the frequency a user connected two other unconnected users, or lied in-between their interactions. As such, it may be viewed as a measure of bridging others and helping communications among interacting participants.
- Closeness centrality: reflects the distance between a user and all other users in the group, a user with high closeness centrality can be easily reachable by all others.

As this article is beyond the discussion of the theory, mathematics and concepts of centrality measures, interested readers can have further details in this article [26].

#### Group level

For each group, we collected the following parameters: The total number of interactions and type (student-student, student-tutor, and tutor-tutor), average in-degree centrality (average in-degree of all group members), average degree centrality (average degree of group members).

### Statistical analysis

Statistics were performed using Paleontological Statistics Software Package for Education and Data Analysis version 3.2. Correlation among variables was performed using the non-parametric Spearman rank correlation test [27].

## Results

### Descriptive statistics

The course included 15 PBL groups; each group had a range of 6 to 12 students with one tutor. There were six discussion forums: five PBL discussion forums and a news forum (for course announcements, data of the news forum were excluded from the study. The course had 2620 interactions, mostly from students to students (89%), students to teacher interactions were 4.9% and teacher to student interactions were 6.15%. The range of interactions in each group was 48 to 332 (mean 216, SD 81.5). The picture was also similar on the course level; students dominated the interactions. Some groups were highly interactive such as groups 150, 144 and 149 and some were not such as groups 146, 140 and 141. A breakdown of each interaction type is presented in the course is presented in Table 1 for group level.

**Table 1:** Summary of interaction types in each group

Group ID	Student to student		Student to tutor		Tutor to tutor		Total	
	n	%	n	%	n	%	n	%
140	70	84.34	3	3.61	10	12.05	83	100
141	72	96	1	1.33	2	2.67	75	100
142	157	83.51	5	2.66	26	13.83	188	100
143	153	95.63	3	1.88	4	2.5	160	100
144	276	99.64	0	0	1	0.36	277	100
145	107	90.68	3	2.54	8	6.78	118	100
146	47	97.92	0	0	1	2.08	48	100
147	84	94.38	2	2.25	3	3.37	89	100
148	198	89.19	12	5.41	12	5.41	222	100
149	277	92.95	7	2.35	14	4.7	298	100
150	271	81.63	23	6.93	38	11.45	332	100
151	163	98.79	1	0.61	1	0.61	165	100
152	51	39.53	56	43.41	22	17.05	129	100
153	241	91.29	12	4.55	11	4.17	264	100
154	164	95.35	1	0.58	7	4.07	172	100
Total	2331	100	129	100	160	100	2620	100

### RQ1: what can SNA visual and mathematical analysis tell about PBL?

SNA can be used to map the relationships among actors, groups (visual analysis) and calculate the mathematical metrics of interactions and actors. The visual analysis was used to explore its role in shedding lights on the individual actors and their groups and how could this help learners or teachers. Furthermore, the mathematical analysis was used to quantify the interactions on the same levels, to demonstrate the utility of quantifications of interactions and how it compares to visual analysis.

### Visual analysis

SNA visual analysis can be used on different levels, in our case three levels are relevant: the whole course level, the PBL group level and individuals’ level. Following is a demonstration of what each level can reveal visually.

#### On course level

**Fig. 1:**
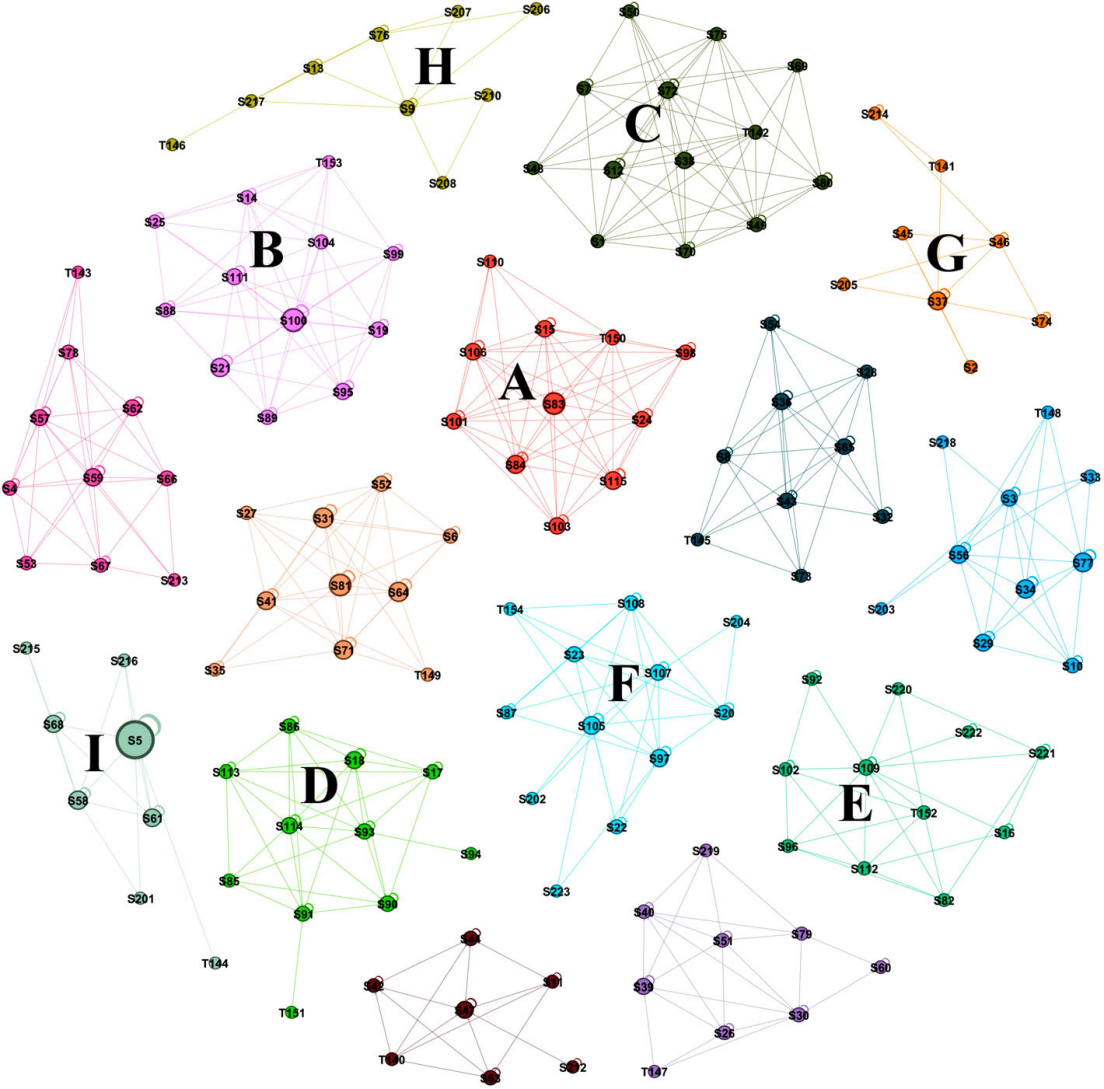
Interactions in all groups. Each circle represents an actor, each arrow represents an interaction, groups were coloured with unique colours to be easy to distinguish

One of SNA biggest advantages is the ability to sum up and map all interactions, as such it offers a bird-eye view of the relational dynamics in a course or a group. In the next Fig. 1, SNA rendered each PBL separately and identified each group, a property known as *finding communities*. This property is useful in finding the group of members who frequently interact with each other. The Figure shows each PBL group and the interactions among participants. Active groups can be recognized; an example is group A, B, and C; the three groups have dense interactions among participants. Groups D, E, and F, have a moderate level of activity; while G and H groups are less active with very few interactions among participants. Using such method may help pick the groups that need attention, a focused review or support.

#### On group level

**Fig. 2:**
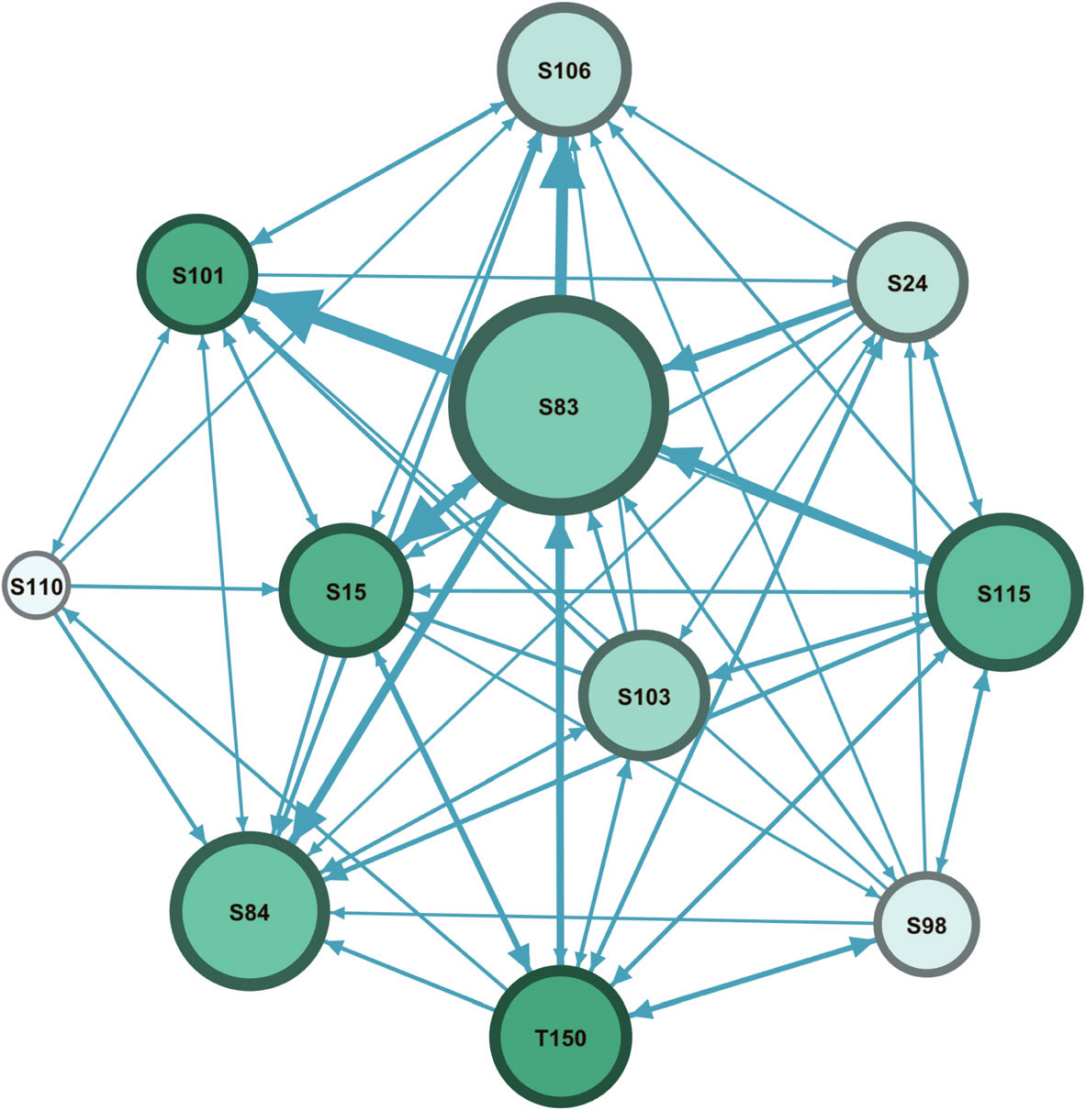
Interactions in group A. The node size was configured as degree centrality to reflect participants’ activity. The edges were configured so that the more frequent the interactions between two participants are, the thicker the line will be. The colour intensity reflects role in moderating interactions

While the insights from the whole course network can be helpful to have a course overview, a focus on each group separately can demonstrate deeper information. In each group, the visual analysis using SNA can reveal the patterns of the interactions occurring in the group and how the students and the tutor perform; which are relevant factors for the evaluation of the PBL process. To demonstrate the potential of SNA and how much insight it can give, we present three Figures, in Fig. 2, group (A) is visually rendered in a sociogram. Regarding participants’ roles, it can be noted that student S83, S84, S15 are the most active students, while S110, S98 are the least active and are relatively isolated. The interactions are mostly occurring among students S15, S101, S84, S106 and the tutor. The role of the tutor who is coded as T150 can also be recognized, the node size indicates a moderate level of activity, and the arrows directions are indications that he received many interactions as well as communicated back with most students, the dark node color is an indication that his role was mostly moderating discussions among students. It is reasonable to say here that the information about the tutor was assuring that that tutor acted as expected from him. Regarding the group, the multiple interactions are an indication of an active group with diverse participation among most participants. To summarize, the picture SNA is rendered in Fig. 2, a picture of an active group with many active students, and few inactive students S110 who might need more attention from the tutor.

**Fig. 3:**
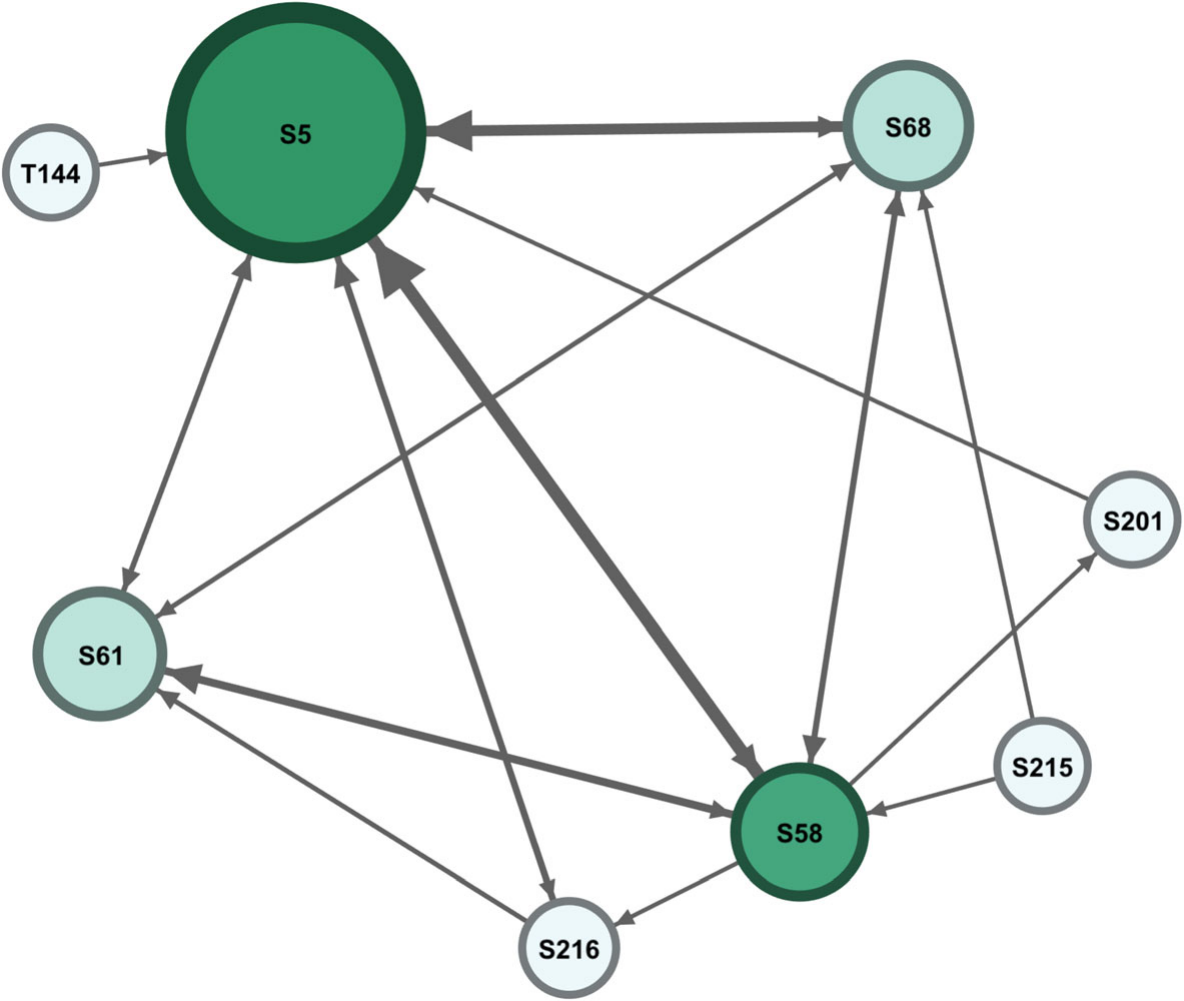
Interactions in group I. The node size was configured as degree centrality to reflect participants’ activity. The edges were configured so that the more frequent the interactions between two participants are, the thicker the line will be. The colour intensity reflects role in moderating interactions

In contrast to the previous group, the next group (I) shows far lower level of activity. However, as shown in Fig. 3, student S5 is the most active participant, less so are students s58, S61 and S68, the remaining students are mostly inactive, the tutor role was very limited and made a single interaction with S5. Apparently, such group requires attention and the tutor might be contacted to play a more active role in the group, to stimulate students and promote interactions.

#### On individual level

**Fig. 4:**
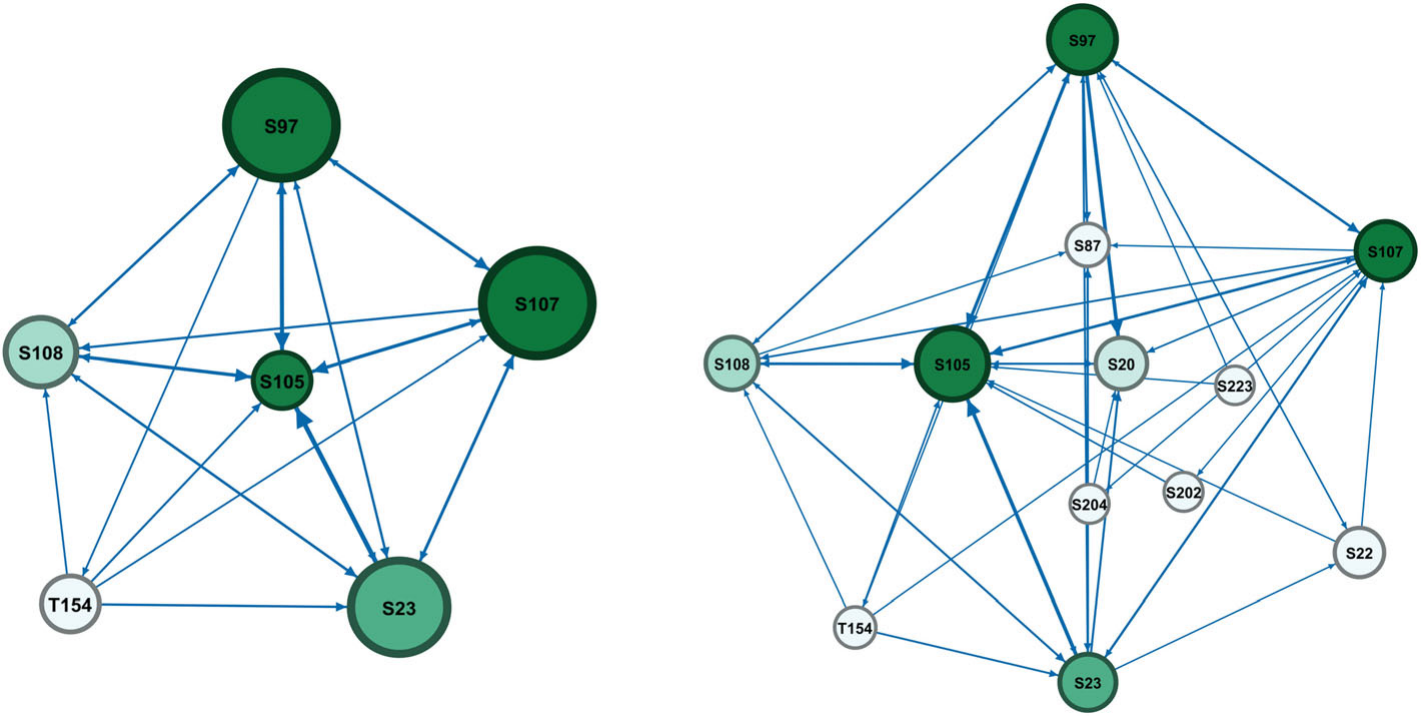
The tutor ego network compared to the whole network showing the tutor has interacted with a limited number of students. The node size was configured as degree centrality to reflect participants’ activity. The edges were configured so that the more frequent the interactions between two participants are, the thicker the line will be. The colour intensity reflects role in moderating interactions

On the individual level, SNA can highlight the network of individual users. For instance, we can have a student of interest that we would like to see his performance. For that situation, we can isolate his network in what is known as an ego network. To demonstrate this, we demonstrate the ego network of the tutor in another group, namely the ego network of the tutor of group I in Fig. 4. Although the group included 11 students, the tutor had only five students who communicated with him. A look at the full network in the side image, we can see that the students who needed help the most (the less active) were not helped, this is a clear example of an opportunity where an improvement can be made, by alerting the tutor to diversify his interactions and focus on the isolated students.

### Mathematical analysis

#### On the course level

Mathematical analysis offers a quantification of interactions and roles which may enable accurate comparison of students and groups. It is also potentially useful as a measurement that can be used to forecast performance. The descriptive properties of participants in our study were as follows: the average outdegree centrality was 17.47, which means each participant has contributed with an average of 3.5 posts/week during the five weeks duration of the course, which is an indication of a relatively interactive course. The centrality measures of information exchange were also acceptable, the closeness centrality normalized score was 0.66, meaning that most students had moderate to high reachability. Same parameters can be obtained for the group and they are presentenced in the next section.

Further details are in Table 2.

**Table 2:** Descriptive statistics of participants

Parameter	n	Mean	SD
Indegree centrality	150	17.47	20.81
Outdegree centrality	150	17.47	18.03
Degree centrality	150	34.93	36.54
Closeness centrality	150	0.66	0.19
Betweenness centrality	150	4.55	7.53

#### On the group and individual level

**Fig. 5:**
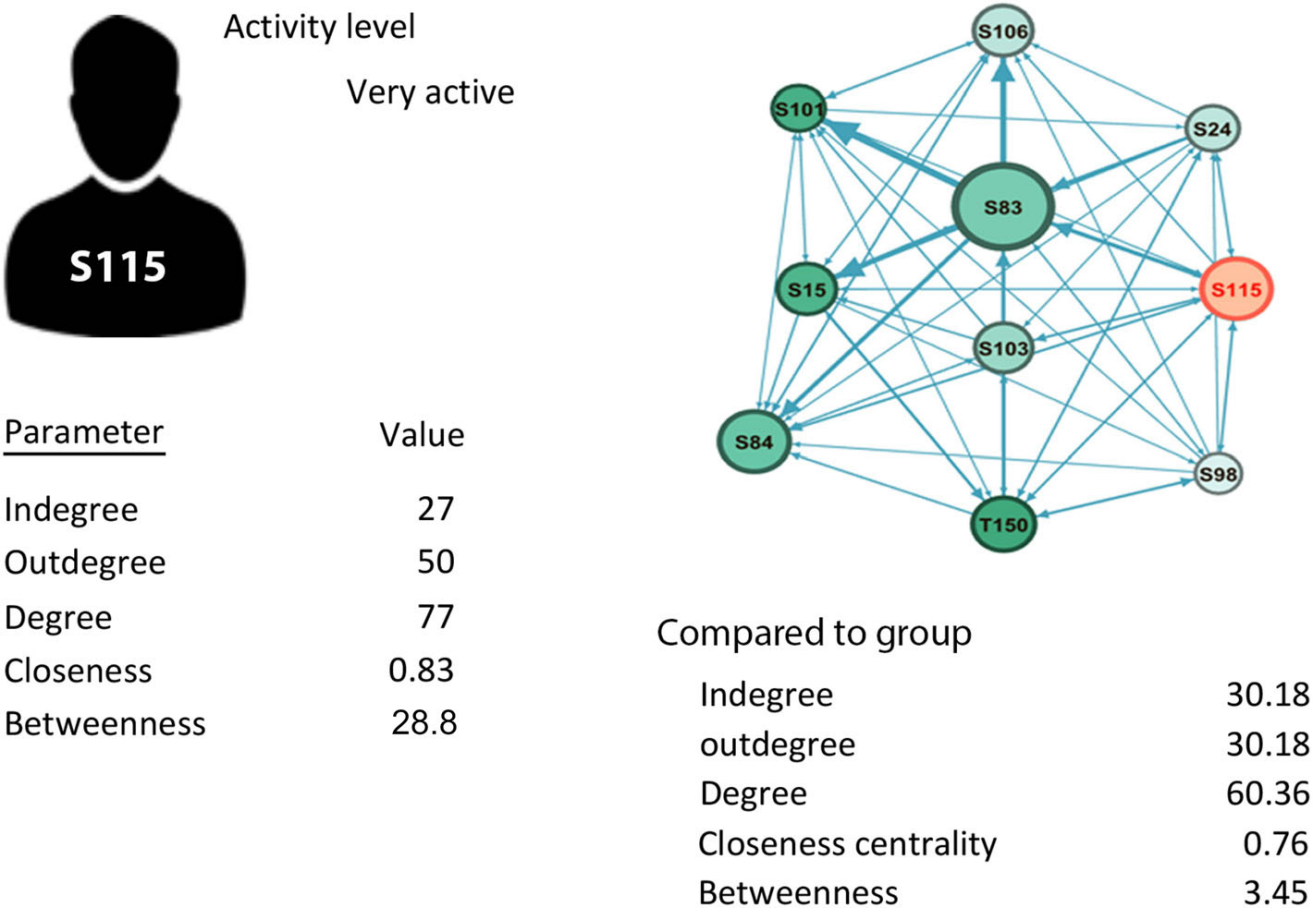
A social network profile model

As a demonstration, the mathematical parameters of a student are summarized in Fig. 5, compared to own group. The student, were actively contributing (outdegree 50) which is average 10 posts every week, received 27 replies (indegree 27), an average of 5.4 posts/week, an indication of the interest of peers to reply to the posts. Furthermore, closeness centrality is high which means he was close in the discussions to all other collaborators. However, betweenness 28.8 centrality is high, an indication that the student has connected and bridged interactions with others. The parameters of the group were also listed on the side to see how the student compared to others in the same group. The parameters were relatively high (high indegree, high outdegree, high closeness and betweenness centralities). One can reach the conclusion that that this was an active student in an active group.

### Social profile

To sum up a student or a user activity, we propose using SNA as a monitoring tool that displays the activity of a use (visual and mathematical) compared to own group for the teachers to use. The social network profile concept we propose can be applied in a learning management system dashboard to show social profile, connections and parameters. To demonstrate this, we chose a student from group A, showing ego network, the activity level and parameters for clarity, we also compare the activity to the group side by side. Comparison to his group will show if his activity level is proportional to colleagues or not and will highlight the other participants’ role. In case there is an unsatisfactory level of activity, one can understand the reason.

### RQ2 do interaction parameters - as measured by SNA - correlate with better performance?

To see which of the SNA factors might correlate with better achievement, we categorized the parameters into three main groups: personal, group and tutor factors. The results indicated that outdegree centrality of a student, being in a group with higher average grade, communicating with the tutors, are the factors most correlated with better learning. The full details of correlation coefficient are displayed in Tables 3.

**Table 3:** Correlation between SNA parameters and achievement

Factors	Correlation with achievement	Sig. (2-tailed)
Student’s factors		
Indegree	0.08	0.38
Outdegree	0.27	0.01
Degree	0.17	0.06
Closeness centrality	0.13	0.16
Betweenness centrality	0.08	0.39
Group’s factors		
Average Degree	0.02	0.84
Average Group grade	0.39	0.00
Number of users	−0.07	0.47
Tutor’s factors		
Teacher-student	−0.02	0.79
Student -teacher	0.22	0.02

## Discussion

This study was done to evaluate the role SNA can be used to evaluate online PBL and offer insights to administrators, tutors, and students. We have looked at the visual analysis and the quantitative interaction statistics. The type of interaction based on the involved participant whether he is a tutor or a student was considered. We also used role classification to try to understand the dynamics of intragroup interactions.

On the quantitative level, SNA interaction analysis offered simple, easy to interpret indicators for the level of activity of students and tutors; these indicators helped identify the active groups, tutors, and students alike. The indicators also helped flag a potentially dysfunctional or inactive group and the inactive student or a disengaged tutor. The SNA measures of role and position in information transfer highlighted qualities that can’t be easily recognized by the interaction parameters (number of posts). For instance, betweenness centrality reflected the role of students in moderating information, connecting the unconnected students and brings them into the discussion. Closeness centrality pointed to students who were not easily engaged in the discussions.

When coupled with visual analysis, it gave further insights into the dynamics of the interactions in the groups, such as who was active, who were the students interacting together and highlighted the isolated students. The role analysis we have performed in our study added another dimension to the utility of SNA. Identifying each role and who plays it enables tutors to understand dynamics in the group, helps motivate discussions and mitigate conflict or prevent dysfunction in interacting groups as well as appreciate different points of view [28, 29].

Studies of interaction analysis in online PBL are scarce, our review of the literature identified one study about the analysis of problem-solving online discussions, which is apparently not precisely online PBL [30, 31]. Another study by Saqr et al. that focused on using social network metrics as proxy leaning analytics indicators to predict performance using machine learning methods and advanced modelling techniques. The importance of our study is that it offers a practical monitoring solution that can be implemented and interpreted by different stakeholders, the insights generated have the potential to improve many aspects of the online PBL, and at times help intervention in a dysfunctional group or a disengaged student [32]. SNA based intervention has proved practical and effective in bringing tangible results that could support teaching and learning in other settings and such methods are similarly applicable in PBL [32].

While content analysis might have added insights about the exchanged information in online PBL, it is resource intensive and requires long, exhaustive work. Since content analysis is usually done manually, it is not a practical choice for automatic monitoring of thousands of interactions. Content analysis has also been criticized for being subjective and difficult to standardize [33–35]. Educational data mining (EDM) offers a potential alternative as it can automatically analyze text in the real-time. Nonetheless, EDM is impractical beyond research purposes since the instruments and frameworks used are difficult to use by teachers. The most challenging hindrance we found in the context of online PBL was the absence of context-specific tools since most of the available tools are from outside the field of education, a problem Alejandro [36] stated as *“Many of the EDM researchers are in reality users of Data mining frameworks and tools. Because student modeling commands the focus of more than half the approaches.”*

While our research was feasible due to the availability of data recorded by the learning management system and the relative ease of obtaining it, a similar approach can be extended to include the face-to-face PBL. The modern voice recognition techniques can accomplish it, since transcribing tools that are becoming more accurate, cheaper and easier to implement. The implementation would require more discussion about what to transcribe, what to store and how to handle this data.

We think that our study offered a demonstration of the potentials of SNA as a tool for interaction analysis of online PBL. We hope that this study can kindle the discussions about the need for automatic monitoring of PBL. We hope that future research can examine, improve or critique our approach. An obvious future approach to online PBL could couple learning analytics methods with SNA methods.

While this study has shed lights on the communicative and relational aspects of the online PBL, it is not without limitations. We recognize the limitation and the need for a reliable content analysis method. An automatic EDM method that is designed to capture the PBL elements and give a credible insight into the content of the discussions. We also recognize that our results might need further confirmation in other contexts to advance our understanding of the process, especially different implementations of online PBL.

## Conclusions

Social network analysis is a practical method that can reliably monitor the interactions in an online PBL environment. Using SNA can reveal important information about the course, such as the general activity and the active groups. On the group level, it renders a more detailed picture about the group and the participants. Thus, helping to identify the active, inactive or isolated students and tutors alike. Using mathematical parameters can also help add insights about the level of activity in a precise way. Furthermore, the insights generated by SNA can be useful in the context of learning analytics to help monitor students’ activity.

## Abbreviations

EDM: Educational Data mining
MCQ: Multiple Choice Questions
MEQ: Modified Essay Questions
PBL: Problem Based Learning
RQ: Research Question
SD: Standard Deviation
SEQ: Short-answer Essay Questions
SNA: Social Network Analysis
SQL: Structured Query Language
